# Enhancement of specialized metabolites using CRISPR/Cas gene editing technology in medicinal plants

**DOI:** 10.3389/fpls.2024.1279738

**Published:** 2024-02-21

**Authors:** Swati Das, Moonhyuk Kwon, Jae-Yean Kim

**Affiliations:** ^1^ Division of Applied Life Science (BK21 Four Program), Plant Molecular Biology and Biotechnology Research Center (PMBBRC), Gyeongsang National University, Jinju, Republic of Korea; ^2^ Division of Life Science, Anti-aging Bio Cell Factory Regional Leading Research Center (ABC-RLRC), Research Institute of Molecular Alchemy (RIMA), Gyeongsang National University, Jinju, Republic of Korea; ^3^ Nulla Bio R&D Center, Nulla Bio Inc., Jinju, Republic of Korea

**Keywords:** CRISPR/Cas, gene editing, metabolic engineering, specialized metabolites, medicinal plants

## Abstract

Plants are the richest source of specialized metabolites. The specialized metabolites offer a variety of physiological benefits and many adaptive evolutionary advantages and frequently linked to plant defense mechanisms. Medicinal plants are a vital source of nutrition and active pharmaceutical agents. The production of valuable specialized metabolites and bioactive compounds has increased with the improvement of transgenic techniques like gene silencing and gene overexpression. These techniques are beneficial for decreasing production costs and increasing nutritional value. Utilizing biotechnological applications to enhance specialized metabolites in medicinal plants needs characterization and identification of genes within an elucidated pathway. The breakthrough and advancement of CRISPR/Cas-based gene editing in improving the production of specific metabolites in medicinal plants have gained significant importance in contemporary times. This article imparts a comprehensive recapitulation of the latest advancements made in the implementation of CRISPR-gene editing techniques for the purpose of augmenting specific metabolites in medicinal plants. We also provide further insights and perspectives for improving metabolic engineering scenarios in medicinal plants.

## 1 Introduction

Plants with therapeutic and pharmacological importance produce great natural products applicable for human healthcare with a high market value in the areas of medicine, antioxidants, essences, perfumes, dyes, insecticides, pheromones, and other high-value natural goods for human healthcare (Yuan et al., 2016). Plants have developed their metabolic systems in response to environmental challenges, resulting a diverse array of specialized metabolites production (Springob and Kutchan, 2009).

Plant-synthesized endogenous organic compounds are classified into two main classifications, namely primary and secondary (specialized) metabolites (Sangwan et al., 2018). In order for organisms to ensure their survival, it is imperative that they must possess essential metabolites, including carbohydrates, proteins, lipids, and nucleic acids (Sangwan and Sangwan, 2014b). Precursors for the specialized metabolites are the primary metabolites. The specialized metabolites confer many adaptive and evolutionary advantages with various metabolic functions and are often associated with plant defense mechanisms (Trethewey, 2004). Plants are the richest source of specialized metabolites, with an estimated 100,000 specialized metabolites in about 50,000 plant species (Pyne et al., 2019; Kaushik et al., 2017; Hussein and El-Anssary, 2018; Yang et al., 2018). Plant-specialized metabolites can be categorized into three primary classifications: terpenes, nitrogen-containing compounds, and phenolics, based on their biosynthetic pathways, and are categorized into volatiles, sterols, carotenoids, glycosides, alkaloids, glucosinolates, and tannins (Bourgaud et al., 2001; Jadaun et al., 2017). Specialized metabolites play a pivotal role in facilitating plants to combat adverse environmental and physiological stresses, unlike the primary metabolites which are essential for plant growth and development (Verpoorte et al., 2002; Singer et al., 2003).

Plant-specialized metabolites are essential for health improvement; they are used for antitumor, antimicrobial, anticancer, and antibiotic, such as bleomycin, pentacyclic terpenearjunolic acid, taxol, and penicillin, respectively (Sangwan et al., 2018). Medicinally significant specialized metabolites found in plants like *Taxus*, *Withania*, *Centella*, *Artemisia*, and *Cymbopogon* include taxol, artemisinin, withaferin, asiaticoside, withanone, madecassoside and essential oil (Sangwan et al., 2004, 2007;Sangwan et al., 2008; Chaurasiya et al., 2012; Sangwan et al., 2014a; Sangwan and Sangwan, 2014b). Traditional uses for these therapeutic herbs date back thousands of years.

**Figure 1:**
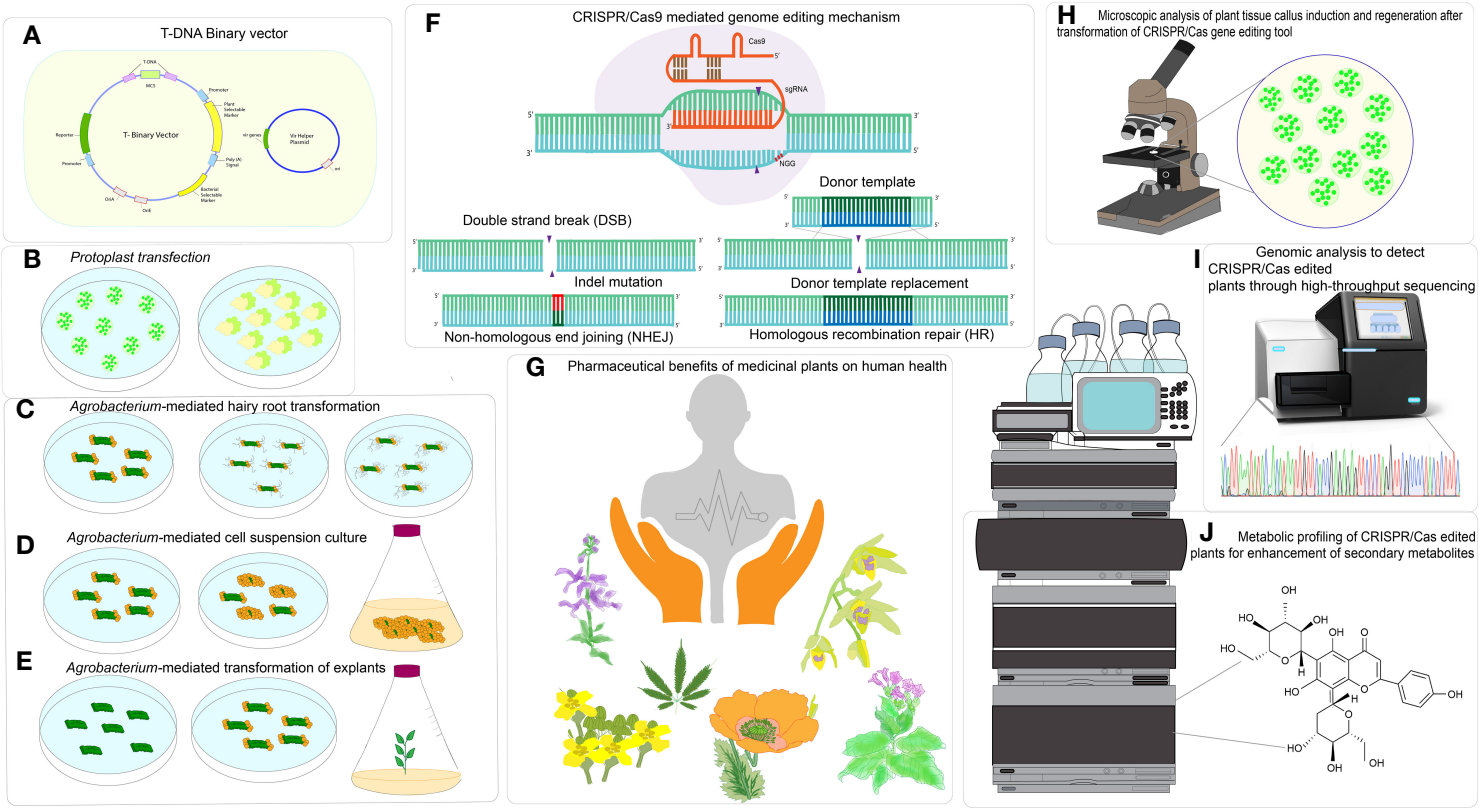
CRISPR/Cas based genome editing in medicinal plants for enhancement of specialized metabolites. **(A)** T-DNA Binary vector, **(B)** Particle bombardment of plasmid or CRISPR ribonucleoprotein (RNP) in protoplast, **(C)** *Agrobacterium*-mediated hairy root transformation, **(D)** *Agrobacterium*-mediated cell suspension culture, **(E)** *Agrobacterium*- mediated transformation of explants, **(F)** CRISPR/Cas-mediated genome editing mechanism, **(G)** Pharmaceutical benefits of medicinal plants on human health, **(H)** Microscopic analysis for analysis plant tissue callus induction and regeneration after transformation of CRISPR/Cas tool delivery**, (I)** Genomic analysis to detect CRISPR-edited plants through High throughput sequencing, **(J)** Metabolic profiling of CRISPR- edited plants for enhancement of secondary metabolites.

In the contemporary age of biotechnology, research on medicinal plant-based bioactive compounds constantly upgrades the information in databases. Also, contributes to the growth of therapeutic drugs, agro-food, agrochemical, and beauty (Hassan, 2012). *Salvia miltiorrhiza*, *Cannabis sativa, Dendrobium officinale*, and *Opium poppy* have effective transformation mechanisms and high-quality reference genomes (Xu et al., 2016; Guo et al., 2018; Gao et al., 2020; Niu et al., 2021). Scientists are finding novel synthetic methods and focusing on mining critical genes for functional gene studies, producing effective compounds, and metabolic network regulation of medicinal plants for improving the quality and breeding superior germplasm. Retrieving genomic data from an ever-expanding variety of plant species and manipulating the genome is now possible thanks to the advancement of better genome engineering systems. These systems allow for precise and effective editing of target-specific genes at predetermined loci within a genome, repurposing the functions of specific targeted regions **Figure 1A**.

Targeted genome editing works by using a sequence-specific nuclease to instigate a DNA double-strand break (DSB) at a target site that repairs by either the donor-dependent homology-directed repair (HDR) pathway or the erroneous non-homologous end joining (NHEJ) pathway repair (Zhu et al., 2020). Early generation sequence-specific nucleases that depend on protein-DNA interactions, such as meganuclease (MN), zinc-finger nucleases (ZFNs), and transcriptional activator-like effector nucleases (TALENs), have adequately contributed to plant gene editing (Puchta et al., 1993; Wright et al., 2004; Christian et al., 2010). However, the impediment arises with their construction because their production necessitates intricate protein engineering. For a pre-determined sequence, nucleic acid cleavage activity performed by the third generation of CRISPR (clustered regularly interspaced short palindromic repeats)/Cas (CRISPR-associated protein) relying on DNA-RNA recognition demonstrates a greater advantage than protein-DNA interaction requiring complex engineered protein (Mali et al., 2013). Thus, CRISPR/Cas9 and its other orthologs or alternative CRISPR/Cas systems can introduce DSBs at specified target sites with little effort and expense. The DNA modifications the genome editing tools induce could be deletions, insertions, or substitutions (Bhatta and Malla, 2020). Due to its affordability compared to herbal medicines and allopathy and synthetic drugs, the global market for plant-derived products is anticipated to gain momentum over the upcoming years with the advancement of modern biotechnology. The growth in the plant-derived medicine market is due to increasing demand and intensive research investments and funding, and hopefully, it will increase rapidly within the next few decades.

This review paper examines the utilization of CRISPR/Cas approach to augment the biosynthesis of specific metabolites in agriculturally significant crops, including wheat, tomato, and rice. We also emphasize medicinal plants that are enriched by natural bioactive compounds necessary for industrial and pharmaceutical purposes, for instance, medical drugs, perfume, and cosmetic industries (Hassan, 2012). Subsequently, our focus shifts towards the plausible application of gene editing techniques for enhancing the biosynthesis of certain natural compounds *in vitro*.

## 2 Utilization of CRISPR/Cas gene editing approach in the plant biosynthetic pathway

### 2.1 The CRISPR/Cas system and how it operates

An adaptive system for phage immunity in bacteria and archaea, CRISPR/Cas, originates from prokaryotes. CRISPR/Cas system allows programmable RNA-guided genetic manipulation, as it is dependent on the recognition of DNA-RNA interaction and pre-determined sequence-specific nucleic acid cleavage binding activity (Zhu et al., 2020). This third-generation genome editing technology has the advantage over the earlier gene editing systems, namely, ZFN (Wright et al., 2004) and TALEN (Christian et al., 2010), because their fabrication requires complex protein engineering, high cost, and lack of versatility for building a multiplex mutation system (Knott and Doudna, 2018; Kim and Kim, 2019; Chen et al., 2019a). The single-guide RNA (sgRNA), which comprises the fusion of a trans-activating RNA (tracrRNA) and a CRISPR RNA (crRNA), is part of the CRISPR/Cas system, derived from type II *Sp*Cas9 (the most widely adopted Cas9 protein from *Streptococcus pyogenes*) and has extensive utilization in various species, including plants. The sgRNA-Cas combined complex binds to the desired DNA sequence, cleaves the DNA, and recognizes a protospacer adjacent motif (PAM) site adjacent to the sgRNA binding site (Jinek et al., 2012). Another popular Cas enzyme, Cas12a, is a class 2, type V, RNA-guided DNA endonuclease having a single nuclease domain that requires a single, smaller gRNA molecule, RNAase activity for processing crRNA, generates staggered DNA ends, and detects T-rich PAM sites (Kadam et al., 2018).

The fundamental mechanism underlying the CRISPR/Cas tool is to achieve DSB at a designated genomic locus and repair broken gaps in DSBs by introducing DNA modifications by the donor-dependent HDR pathway or the erroneous NHEJ pathway (Guo et al., 2022). By reconnecting the two ends of the DSB, erroneous NHEJ introduces inaccurate short nucleotide insertions and deletions (indels) into the targeted regions (such as genes and promoters), causing frameshift alterations that interfere with the original structure and function (Jacobs et al., 2015). In juxtaposition to DSBs repaired by NHEJ, the repairs facilitated by HDR involve the precise insertion or replacement of a predefined nucleotide sequence using external homologous donor templates (**Figure 1F**).

Due to the expeditious progression of the CRISPR/Cas technology, it has undergone significant developments that surpass traditional targeted mutagenesis mediated by DSBs. These advancements encompass various gene editing tools such as base editors (Kim, 2018), high precision editing achieved through HDR and prime editors (Anzalone et al., 2019; Huang and Puchta, 2019), as well as transcriptional regulation (Mahas et al., 2018). Furthermore, the CRISPR/Cas system possesses the capacity to alter the expression of genes through the manipulation of transcriptional regulatory elements, including promoters, enhancers, terminators, and other related components (Zhang et al., 2019b).

Base editing and prime editing approaches do not rely on conventional CRISPR/Cas mechanisms for repairing a DSB in the gene sequence. The base editing tools are classified into cytosine base editors (CBEs) and adenine base editors (ABEs). These base editing tools comprise either a nicked Cas9 (nCas9) or catalytically inactivated Cas9 (dCas9) coupled with a particular deaminase. The deaminases induce transitory alterations in DNA by converting C•G to T•A or A •T to G •C, depending on their unique functions (Komor et al., 2016). Moreover, the prime editing tools comprised two main components: a fusion protein that includes Cas9 nickase (H840A) and reverse transcriptase, in addition to a second component called prime editing guide RNA (pegRNA). The Cas9 nickase variant (H840), consisting of a RuvC functioning domain, introduces nick to the non-target DNA strand. Reverse transcriptase then works with a pegRNA template to modify the necessary DNA (Anzalone et al., 2019). The primer editor tool has evolved into a sophisticated platform for facilitating accurate base substitution, DNA deletion, and DNA insertion.

The CRISPR/Cas system also involves in gene regulation via two main methods: transcriptional activation, referred to as CRISPRa, and transcriptional repression, known as CRISPR interference or CRISPRi (Qi et al., 2013; Chavez et al., 2015; Konermann et al., 2015). Moreover, recent advancements have resulted in innovative CRISPR-based methodologies aiming to fuse of epigenetic modifiers with dCas9. These strategies have been undertaken to modify the epigenome to attain precise alterations in DNA methylation and histone modifications (Abudayyeh et al., 2017; Gootenberg et al., 2017; Zhang et al., 2019b).

### 2.2 Current status of plant metabolic engineering in CRISPR era

The methodologies employed in the domain of metabolic engineering in plants can be categorized into two primary objectives: (1) nutritional quality improvement or biofortification, and (2) plant synthetic biology (Camerlengo et al., 2022). Enhancing the nutritional composition of food, often known as biofortification, is a critical objective in applied plant biology research. According to the World Health Organization (UN Report, 2022), 828 million individuals across the globe, particularly in underdeveloped and emerging nations, experience malnutrition due to inadequate nutrient intake. Considering the profound influence of nutrition on human welfare, implementing the CRISPR/Cas tool in biotechnology exhibits the potential for augmenting the nutritional profile of crop plants (Mora-Vásquez et al., 2022). Recently, several desired outcomes in crop plants engineered for enhancing specialized metabolites have been standardized and accustomed through targeted alteration of essential biosynthetic or metabolic processes within the genome, as outlined in **Table 1**. The understanding of current knowledge in CRISPR/Cas-mediated plant metabolic engineering in crop plants serves as a prototype for strategies applicable to increasing medicinally-valued metabolites in medicinal plant with limited information.

**Table 1:** Application of CRISPR/Cas gene editing technology for enhancement of specialize metabolites in plants.

Plant species	Target gene	Nutritional/Pharmaceutical Improvement	Targeted metabolic Pathway	Strategy	Result	Reference
Tomato	LCY-E, SGR1, Blc, LCY-B1, LCY-B2	Vitamin A enhancement; Lycopene accumulation	CRISPR/Cas9 mediated Knockout Carotenoid metabolic pathway	A bidirectional approach was adapted with the aim of augmenting lycopene production while concurrently inhibiting the conversion of β- and α-carotene from lycopene.	Lycopene concentration in tomato fruit increased by a fold of 5.1, subsequent after implementing multiplex genome editing	(Li, X. et al., 2018)
Rice	CrtI and PSY	Vitamin A enhancement; beta- carotene	CRISPR/Cas9 mediated 5.2kB sized insertion without selectable marker. Carotenoid metabolic pathway	The 5.2 kB-sized cassette had the coding sequences of two genes, specifically *SSU-crt*I and *ZmPsy*	Increased carotenoid levels in the rice seeds	(Paine et al., 2005; Dong et al., 2020, 2020)
Tomato	SlGAD2, SlGAD3, SlGABA-Ts, SlCAT9 and SlSSADH	GABA accumulation	CRISPR/Cas9-mediated C- terminal deletion of Calmodulin domain of GABA shunt pathway	Introducing pre-mature stop codon and gene disruption.	Increased GABA content in tomato fruit and leaves.	(Nonaka et al., 2017; Li, R. et al., 2018)
Rice	OsGAD3	GABA accumulation	CRISPR Cas9 mediated C- terminal deletion of Calmodulin domain of GABA shunt pathway	Introducing pre-mature stop codon	Increased GABA content in rice seeds	(Akama et al., 2020)
Tomato	7-dehydrocholesterol (7- DHC)	Enriched Vitamin D by duplicate SGA biosynthetic pathway	CRISPR/Cas9-mediated knockout of SGA biosynthesis	7-dehydrocholesterol reductase (Sl7-DR2) facilitates the conversion of 7-DHC to cholesterol, which is essential for the production of α-tomatine in both tomato leaves as well as fruit	. To enhance the provitamin D3 levels in tomatoes through biofortification, the activity of Sl-DR2 was disrupted, leading to the buildup of 7-DHC	(Li, J. et al., 2022)
Tomato	R2R3MYB, Anthocyanin Pigment 1 (PAP1, MYB75), DFR, F3H, and F3’H and genes *ANT1*, *AN2*-like, *AN2*	Anthocyanin accumulation	CRISPR/Cas9- mediated TF gene knockout	TFs family member gene targeting	Study of functional role of TFs in anthocyanin biosynthesis pathway	Riaz et al., 2019; Wang et al., 2019
Lettuce	LsGGP2 uORF	Improvement of antioxidants, oxidative stress tolerance and 150% increase in ascorbate content	CRISPR/Cas9 mediated Knockout of SGA biosynthesis			(Si et al., 2020; Zhang, et al., 2018b)
Lettuce	CPT3	Natural Rubber	Rubber biosynthesis			(Kwon et al., 2023)
*Brassica napus*	BnITPK	Free of Phytic acid	35% decrease in phytic acid by CRISPR Cas9 mediated gene editing			(Sparvoli and Cominelli, 2015)
Wheat	TaIPK1	Free of Phytic acid	Reduced levels of Phytic acid, increase Fe 1.5-2.1 fold to 1.6 to 1.9 fold			(Sashidhar et al., 2020)
Wheat	TaASN2	Free of Asparagine	90% Reduction in asparagine			(Raffan et al., 2021)

Rice and tomato have been enriched with carotenoid and lycopene accumulation by targeting the carotenoid biosynthetic pathway using CRISPR/Cas-mediated multiplexing knockout by NHEJ repair, and targeted homologous recombination inducing DSBs, or targeted insertion of DNA fragments without selectable markers (Dong et al., 2020; Li, X. et al., 2018; Globus and Qimron, 2018; Hayut et al., 2017; Shlush et al., 2021).

CRISPR/Cas9 multiplex genome editing utilized to alter five genes from the lycopene metabolic pathway, namely lycopene E-cyclase (*LCY-E*), *SGR1*, lycopene β-cyclase 1, β-lycopene cyclase (*Blc*), and *LCY-B2*. It reported that the lycopene concentration in tomato fruit increased by a factor of 5.1 after implementing multiplex genome editing (Li et al., 2018). Moreover, CRISPR/Cas9, was also employed to induce targeted homologous recombination (HR) in somatic cells, aimed at investigating the involvement of two particular genes, carotenoid isomerase (CRTISO) and phytoene synthase (PSY1), in the carotenoid biosynthesis pathway of tomato plants (Filler Hayut, Melamed Bessudo, and Levy 2017; Shlush et al., 2021). Another study in rice plants exhibited enhanced carotenoid levels in the seeds effectively produced by a targeted insertion of a 5.2 kb-sized carotenoid biosynthetic cassette without a selectable marker into the rice genome. The 5.2 kB-sized cassette had the coding sequences of two genes, specifically *SSU-crt*I and *ZmPsy* (Paine et al., 2005; Dong et al., 2020).

Another noteworthy example is the CRISPR/Cas9-mediated genetic alteration to increase the levels of the specialized metabolite, GABA (γ-aminobutyric acid), a non-proteinogenic amino acid, in prominent crops, such as rice and tomato. GABA, known for its hypotensive properties, with increased GABA levels in tomato fruits or rice grains, has the potential to confer advantageous effects on human health by reducing blood pressure. In their study, Nonaka et al. (2017) and Akama et al. (2020) focused on introducing a premature stop codon in close proximity to the autoinhibitory Ca2+/calmodulin binding domain (CaMBD) of glutamate decarboxylase (GAD) genes to enhance the GABA accumulation in tomato fruit, and rice, respectively (Nonaka et al., 2017; Akama et al., 2020). Li et al. (2018) devised an alternative way to strategize a multiplex CRISPR/Cas strategy enabling efficient targeting of five distinct genes associated with the GABA shunt pathway, which included GABA-TP1, GABA-TP2, GABA-TP3 (also referred to as TP-pyruvate-dependent GABA transaminase), CAT9, and SSADH (Koike et al., 2013; Snowden et al., 2013; Bao et al., 2015; Li et al., 2018).

The prevalence of nutritional deficiencies, such as vitamin D deficiency, is a significant concern on a global scale. Deficiency of vitamin D is associated with increased susceptibility to cancer, neurocognitive decline, and impaired skeletal development. Li et al. (2022) successfully attempted to increase 7-dehydrocholesterol (7-DHC) levels, often referred to as provitamin D3, in tomatoes, effectively with the employment of CRISPR/Cas9. A supplementary metabolic pathway related to steroidal glycoalkaloids (SGA) biosynthesis in tomato, wherein a distinct isoform known as 7-dehydrocholesterol reductase (Sl7-DR2) facilitates the conversion of 7-DHC to cholesterol, which is essential for the production of α-tomatine in both tomato leaves as well as fruit. To enhance the provitamin D3 levels in tomatoes through biofortification, the activity of Sl7-DR2 was disrupted, leading to the buildup of 7-DHC (Jäpelt and Jakobsen, 2013; Sonawane et al., 2016; Li, J. et al., 2022).

The development of purple pigmentation in plant tissues is greatly influenced by anthocyanin accumulation, a unique plant metabolite closely linked to enhanced resistance to herbivory, fungal pathogens, bacterial infections, and heavy metal-induced stressors. Anthocyanin, a group of water-soluble flavonoids, plays a significant role in distinct physiological processes, namely gastrointestinal absorption, bowel function, and cardiovascular and neurological diseases (Khusnutdinov et al., 2021). R2R3MYB predominantly controls anthocyanin biosynthesis regulation, the Anthocyanin Pigment 1 (PAP1, MYB75) gene family, DFR, F3H, and F3’H and genes *ANT1*, *AN2*-like, *AN2* (unique to *Solanum lycopersicum*), making them desirable target for CRISPR/Cas gene editing for anthocyanin accumulation (Riaz et al., 2019; Wang et al., 2019).

The manipulation of endogenous upstream open reading frames (uORF) inside plant genomes allows for controlling the regulation of mRNA translation originating from primary open reading frames (ORFs). According to Zhang et al. (2018a) and Si et al. (2020), the editing of uORFs, in the *LsGGP2* gene, a crucial enzyme in the production of Vitamin C, led to a significant enhancement in oxidative stress tolerance and ascorbate content, with a notable rise of 150% (Zhang et al., 2018b; Si et al., 2020).

The prospect of utilizing CRISPR/Cas9-mediated gene editing in plant metabolic engineering expands and unfolds mechanisms of novel biosynthetic pathways in plants. For instance, the knockout variants of *LsCPT3*, Kwon et al. (2023) optimized by the CRISPR/Cas9 tool in lettuce plants. In lettuce laticifers that have undergone CPT transformation, it is possible to make natural rubber with a molecular weight (Mw) exceeding 1 million Daltons. Conversely, it noted that indigenous golden rods could produce natural rubber (NR) with a molecular weight of approximately 0.1 million Daltons (Da). This characteristic provides significant insights into the mechanism of biosynthesis of natural rubber in plant life (Kwon et al., 2023). Recent advancements in the metabolic engineering of agricultural plants have facilitated the augmentation of specialized metabolite levels that have provided the discovery of novel roles within biosynthetic pathways and the enhancement of nutritional or commercial value in these plants.

## 3 Utilizing CRISPR/Cas gene technology in medicinal plants to increase the production of specialty metabolites

Medicinal plants provide bioactive specialized metabolites serving as valuable derivatives for the commercial therapeutics industry **Figure 1G**. Improvements in the synthesis of these medicinally valuable specialized metabolites obtained in the decades before the CRISPR/Cas era. The non-CRISPR/Cas technology involved in genetic manipulation by application of transformation techniques, including gene silencing, gene stacking, and overexpression (Wilson and Roberts, 2014), with a concurrent aim to reduce production expenses, although not as precise manipulation as the CRISPR/Cas system. The application of metabolic engineering for augmenting the synthesis of specialized metabolites and other natural products in medicinal plants, utilizing non-CRISPR/Cas genetic manipulation and CRISPR/Cas-mediated genome editing technology, is exemplified in **Tables 2**, **3**.

**Table 2:** Application of non-CRISPR/Cas technology for the enhancement of specialized metabolites in medicinal plants.

Plant species	Gene manipulation technique	Specialized metabolites	Target gene/Purpose	Reference
*Papaver bracteatum*	Endogenous expression of CodR, a crucial enzyme for converting thebaine to morphine	Elevated codeine levels by eleven-fold and morphine levels with marginal increase of 0.28 percent	Upregulated codeinone reductase (*CodR*)	Sharafi et al., 2013
*Scopolia lurida*	Introduction of external foreign enzyme	Accumulation of scopolamine	Overexpression of hyoscyamine 6β-hydroxylase (*Hn*H6H) from *Hyocyamus niger*	Lan et al., 2018
*Catharanthus roseus*	Overexpression of transcription factor of a specific metabolic pathway	Elevated tabersonine, an indole alkaloid for vinblastine production	Overexpression of *ORCA4* TF	Paul et al., 2017
*Artemisia annua*	RNAi	*AaC4H* RNAi lines exhibited an elevation of trans-cinnamic acid content and reduction in p-coumaric acid levels, salicylic acid (SA) and artemisinin	Gene silencing of cinnamate-4-hydroxylase (*AaC4H*) responsible for the conversion of trans-cinnamic acid (CA) to p-coumaric acid (COA)	Kumar et al., 2016
*Artemisia annua*	RNAi	Accumulation of alkaloid, salutaridine	Suppression of specific gene responsible for expressing the enzyme salutaridinol 7-O-acetyltransferase (*SalAT*)	Allen et al., 2008
*Artemisia annua*	RNAi	Enhancing artemisinin level by 3.14-fold	Gene silencing of squalene synthase (*SQS*), that competes with artemisinin pathway	Zhang et al., 2009
*Catharanthus roseus*	RNA-sequencing	Construction of a database ‘CathaCyc’, with metabolic pathway-related information	Facilitate visualization and interpretation of transcriptomic data	Strickler et al., 2012
*Ophiorrhiza pumila*	Metabolomics	Metabolomic analysis of suspension culture and hairy roots	Distinguish between potential genes regulating monoterpene indole alkaloids and anthraquinones	Higashi and Saito, 2013

**Table 3:** Application of CRISPR/Cas gene editing technology for enhancement of specialized metabolites.

Plant species	Specialized metabolites	Target gene	CRISPR/Cas gene editing strategy	Target site; sgRNA and Cas9 Promoter	Transformation method	Mutation Rate; Gene- editing improvement	Reference
*Salvia miltiorhiza*	Laccase (multi-copper containing glycoproteins)	SmLACs (SmLAC7 and SmLAC20)	Targeting conserved domains to knockout multiple genes of laccase family; functional study	First exon;AtU6::sgRNA; CaMV35S::SpCas9	Hairy roots	Accumulation of lignin and phenolic acid	(Zhou et al., 2021)
*Salvia miltiorhiza*	Phenolics compounds	bZIP	Knockout lines; functional study to prove negative regulator	First exon sequence od bZIP2; AtU6::sgRNA; CaMV35S::hSpCas9	Hairy roots	40% mutation rate, elevated phenolic acid, PAL (phenylalanine ammonia-lyase)	(Shi et al., 2021)
*Salvia miltiorhiza*	Phenolics compounds RA and LAB	RAS	Knockout	ORF;AtU6::sgRNA; OsU3::sgRNA CaMV35S::hSpCas9	Hairy roots	50% in AtU6::sgNA, Lithospermic androsmaniric acid content were reduced	(Zhou et al., 2018)
*Salvia miltiorhiza*	Tanshinones	CPS1	Multiplex knockout	First,fourth, eleventh exon. AtU6::sgRNA1,sgRNA2, sgRNA3; CaMV35S::hSpCas9	Hairy roots		(Li et al., 2018)
*Atropa belladona*	Anticholinergic tropane alkaloids (TAs), hyoscyamine, scopolamine anisodamine.	AbH6H	Pre-mature stop codon	Second exon of ORF;AtU6::sgRNA; CaMV35S::SpCas9	Tissue culture cotyledon	63.6% Mutation rate;3.68-4.21 Fold increase in Hyoscyamine	(Zeng et al., 2021)
*Dendrobium officinale*	Polysaccharides, bibenzyls, essential oil	C3H, C4H, 4CL, CCR and IRX	Frameshift	OsU3::sgRNA; CaMV35S::pcoCas9	Hairy roots	10%-100% editing efficiency	(Kui et al., 2017)
*Nicotiana tabacum*	Plant-derived glycoprotein immune-responsive residue	XylT and FucT	Multiplex knockout	1^st^ exon and 3^rd^ exon; U6::sgRNA, 35S::pcoCas9	Tissue culture	No detectable FucT and XylT residue.	(Mercx et al., 2017)
*Nicotiana tabacum*	Nicotine-free	BBl	Knockout	AtU6::sgRNA; 35S::SpCas9	Tissue culture	99.6% Nicotine free	(Schachtsiek and Stehle, 2019)
*Cannabis sativa*	THC-free (proposed)	CsPDS; THCA	Multiplex Knockout	Exon6, multiple sgRNA	Tissue culture	Albino phenotype	(Zhang et al., 2021)
Comfrey (*Symphytum officinale*)	Homospermidine toxic free	HSS	Knockout	AtU6::sgRNA; 35S::Cas9	Hairy roots	Reduced level of homospermidine	(Zakaria et al., 2021)
Opium Poppy (*Papaver somniferum*)	Benzylisoquinoline	4’OMT2	Knockout	AtU6::sgRNA; 35S::hCas9	Agroinfiltration	S- reticuline for benzylisoquinoline alkaloids production	(Alagoz et al., 2016)
*Dioscorea zingiberensis*	Diosgenin	Dzfps	Frameshift	First exon; OsU3::sgRNA; 35S::SpCas9	Hairy roots	Decreased squalene level	(Feng et al., 2018)
*Taraxacum kok-saghyz*	Natural rubber	1-FFT	Knockout	AtU6::sgRNA; 35S::pcoCas9	Hairy root	Natural rubber synthesis	(Iaffaldano et al., 2016)

### 3.1 Current status of enhancement of specialized metabolites through non-CRISPR/Cas technology in medicinal plants

Utilizing biotechnological applications to enhance specialized metabolites in medicinal plants needs the characterization and identification of genes regulating an elucidated pathway. It is crucial to consider the interplay of components within the metabolic pathways during specific specialized metabolite biosynthesis, including its interactions with enzymes, gene regulation, sub-cellular localization, and epigenetic regulation (Mora-Vásquez et al., 2022). Advanced biotechnological approaches, such as proteomics and functional genomics, help decipher genes that encode their corresponding regulatory enzymes. The term “gene manipulation strategies” refers to a wide range of methods, including the up-or down-regulation of particular metabolic pathway genes or the enzymes responsible for controlling the rate-limiting steps, multi-gene stacking within the same chromosomal vicinity, targeting or blocking of branching pathways that interfere with the biosynthesis of the desired active product, alteration of transcription factor expression levels that directly or indirectly regulate multiple genes within a biosynthetic pathway, and introducing foreign genes. Therefore, enhancing the accumulation of medicinally valuable specialized metabolites can be achieved by increasing the abundance of these metabolites within the plant system or by augmenting the metabolic flux of a particular pathway using advanced molecular tools (Sanchez and Demain, 2008).

The non-CRISPR/Cas genetic engineering techniques, which involve augmenting the gene expression of a catalyzing enzyme, precursor, or product within a metabolic pathway, can be bifurcated into three distinct methods. To begin with, the endogenous expression of crucial enzymes that regulate the rate of various intricate reactions in a pathway, such as methylation, condensation, isomerization, glycosylation, and acylation, has the potential for an augmented buildup of specialized metabolite accumulation in the plant system (Kulkarni, 2016). One example is *Papaver bracteatum*, a botanical species known for its therapeutic properties characterized by a significant presence of thebaine, but exhibiting relatively lower levels of codeine and morphine. The upregulation of codeinone reductase (*CodR*), the pivotal enzyme for converting thebaine to morphine, leads to a significant elevation in codeine levels by eleven-fold and a marginal increase of 0.28 percent D.W. in morphine content in genetically-engineered hairy roots (Sharafi et al., 2013). Secondly, an alternative strategy is introducing an external foreign enzyme derived from a distinct plant species, which may demonstrate enhanced catalytic efficacy in the conversion process toward the targeted specialized metabolite. The overexpression of the exogenous gene hyoscyamine 6β-hydroxylase (*Hn*H6H) from *Hyocyamus niger* in hairy root cultures of *Scopolia lurida* led to a substantial ten-fold enhancement in the accumulation of scopolamine in comparison to the endogenous overexpression of the gene *Sl*H6H (Lan et al., 2018). The third method is to overexpress any transcription factor regulating the gene transcription of a specific metabolic pathway. The upregulation of one transcription factor has several probabilities to modify multiple interconnected genes of a biosynthetic pathway; also cis-regulatory elements that interact with the DNA-binding domain of transcription factors can have an impact on activating or suppressing related target genes (Mahjoub et al., 2009; Samad et al., 2017). Out of five transcription factors belonging to AP2/ERF TFs in *Catharanthus roseus*, namely, *ORCA2* to *ORCA6* that regulates terpenoid indole alkaloids (TIAs), the upregulated genes associated with the indole and seco-iridoid pathways occur when the *ORCA4* TF is overexpressed and elevates the level of tabersonine, an indole alkaloid crucial for vinblastine production (Paul et al., 2017; Singh et al., 2020) (**Table 2**).

Another gene modification approach involves the accumulation of intermediate precursors in a metabolic pathway by silencing subsequent gene expression in that particular pathway (Sinha et al., 2019). Gene silence refers to inhibiting gene expression, which can occur at either the transcription or translation level. Various methods have been developed to achieve gene silencing, including RNA interference (RNAi), miRNA, and Virus-induced gene silencing (VIGS) (Singh et al., 2018; Li et al., 2015; Ossowski et al., 2008; Liu et al., 2017).

RNAi type of suppression or post-transcriptional gene silencing is an approach with which a particular mRNA gets degraded, associated with double-stranded DNA. Medicinal plants, for instance, *Artemisia annua*, *Catharanthus roseus*, *Nicotiana tabacum*, *Panax ginseng*, *Panax quinquefolius*, *Papaver somniferum*, *Withania somnifera* have been engineered with an RNAi approach (Sinha et al., 2019).to suppress the expression of a gene accountable for the degradation of a desired metabolite (Sinha et al., 2019). The gene silencing methods experimented on cinnamate-4-hydroxylase (*AaC4H*) in *Artemisia annua* are responsible for the conversion of trans-cinnamic acid (CA) to p-coumaric acid (COA) in the phenylpropanoid/lignin biosynthetic pathway exhibited an elevation of trans-cinnamic acid, salicylic acid (SA), artemisinin content and reduction in p-coumaric acid levels (Kumar et al., 2016). In their study published in 2008, Allen et al. documented the accumulation of a hitherto unidentified alkaloid, salutaridine, through the use of RNA interference (RNAi) to repress a specific gene responsible for expressing the enzyme salutaridinol 7-O-acetyltransferase (*SalAT*) (Allen et al., 2008). Suppression of critical genes from a competitive pathway can also accumulate a preferred specialized metabolite. Zhang et al. (2009) worked on enhancing the artemisinin level in *A. annua* by utilizing the hairpin-RNA-mediated RNAi method to modify squalene synthase (*SQS)*, an adherent member of the sterol biosynthesis pathway that also competes with the artemisinin pathway (Zhang et al., 2009) (**Table 2**). RNAi works at the post-transcriptional level to silence the expression of a gene. However, it does not eliminate the function of the targeted gene, analogous to any gene editing tool that permits precision editing within the genome with minimal off-target effects (Barrangou et al., 2015; Alagoz et al., 2016; Kui et al., 2017). The first- and second-generation gene editing tools available in the pre-CRISPR period included MNs, ZFNs, and TALENs (Mani et al., 2005; Bogdanove and Voytas, 2011; Deng et al., 2012). However, these previously designed pre-CRISPR era genome editing tools have limitations in targeting specific genome sequences because they require two distinct protein hybrid configurations, which scarcely recognize the existing regions flanking the target DNA (Kim and Chandrasegaran, 1996; Li et al., 2011). Thus, ZFN and TALENs are tedious to design, less specific, and time-consuming. The application of ZFN and TALEN for genome editing was more substantial in model crop plants than in medicinal plants.

High-throughput sequencing-based ‘omics’ approaches are crucial for identifying and characterizing of novel genes within an unrecognized pathway once a reference genome is accessible (Wilson and Roberts, 2014). This is in addition to exploring genome editing tools in metabolic engineering. Researchers can employ quantitative trait loci (QTL) and genome-wide association studies (GWAS) to ascertain the potential genes accountable for particular phenotypes. In instances where genomic data is lacking for a given species, transcriptomic analyses can be utilized to identify potential biosynthetic pathway genes using differential expression studies. For example, the authors utilized RNA-sequencing data from *Catharanthus roseus* to construct a database with metabolic pathway-related information called CathaCyc. This database was developed to facilitate the visualization and interpretation of intricate transcriptome data (Strickler et al., 2012). The incorporation of data derived from metabolomic and proteomic investigations further enhances comprehension of transcriptome studies. In a study by Higashi and Saito, 2013, metabolomic analysis was performed on both suspension cultures and hairy roots of *Ophiorrhiza pumila*. These cultures were shown to accumulate specialized metabolites (Higashi and Saito, 2013). Through this analysis, the researchers could distinguish between potential genes in regulating the biosynthesis pathway of monoterpene indole alkaloids and anthraquinones (**Table 2**). Next-generation sequencing (NGS) methods and inventive CRISPR/Cas-based breeding tools have emerged as superior alternatives to previously employed protein-engineered gene editing tools namely, ZFNs and TALENs. The CRISPR/Cas editing system shows prospect of being a highly practical toolkit for molecular engineering in medicinal plants, especially regarding the augmentation of the specialized metabolite profiles (Niazian, 2019) (**Figures 1I, J**).

### 3.2 Employment of CRISPR/Cas gene technology for enhancement of specialized metabolites in medicinal plants

In the contemporary period of CRISPR/Cas-mediated gene editing, there is a growing emphasis on utilizing and refining of this technology to augment the specific metabolite profiles in medicinal plants. The CRISPR/Cas9 editing tool has proven to be remarkably constructive in the genetic modification of both crops and prototype organisms. It can induce a wide variety of targeted genetic modifications, including frameshift and premature termination codon, gene substitution, site-specific single-gene knockout, fragment deletion, gene substitution, and targeted gene insertion. Characterizing the phenotypic effects of genetic mutants is a methodical way to establish the functional significance of a gene that regulates the synthesis of a specific metabolite in a biosynthetic pathway. **Table 3** conceptualizes the application of CRISPR/Cas-mediated gene editing techniques in medicinal plants.

#### 3.2.1 CRISPR/Cas-mediated targeted mutagenesis/knockout in medicinal plants

In medicinal plants, CRISPR/Cas9-based gene disruption techniques for targeted mutagenesis have been widely applied for the enrichment of pharmaceutically beneficial specialized metabolites or reducing the levels of toxic metabolites. For instance, the modification of the *hyoscyamine 6β- hydroxylase* (*AbH6H*) gene in *Atropa belladonna* L. generates edited plants that lack anisodamine and scopolamine compounds (Zeng et al., 2021). *Atropa belladonna* is a noteworthy botanical specimen that assumes a pivotal function synthesizing of anticholinergic tropane alkaloids (TAs), including hyoscyamine, scopolamine, and anisodamine. These compounds possess considerable commercial worth within the pharmaceutical sector (Gieger and Hesse, 1833, Lossen et al., 1864). Hyoscyamine is found to have therapeutic value in treating arrhythmias and organophosphate poisoning. On the other hand, anisodamine has shown promise in managing of gastrointestinal colic and vascular spasms. Additionally, scopolamine is found to be effective in alleviating symptoms of motion sickness. Hyoscyamine is a prominent constituent of TAs, while anisodamine and scopolamine, which are derived from hyoscyamine, are considered minor constituents (Viraliyur Yun and Yamada, 1992; Poupko et al., 2007; Ramaswami and Thangavelu, 2015). Therefore, due to their similar structures, hyoscyamine’s independent separation and purification from its two derivatives have become challenging and cost-inefficient from *A. belladonna* raw extracts. In their 2021 publication, Zeng et al. provided evidence that a bifunctional dioxygenase, *hyoscyamine 6β-hydroxylase* (*H6H*), is responsible for the enzymatic conversion of hyoscyamine to anisodamine via 6β-hydroxylation, followed by the subsequent epoxidation of anisodamine to scopolamine (Hashimoto and Yamada, 1986; Zeng et al., 2021). The authors employed CRISPR/Cas9-targeted mutagenesis techniques to modify *H6H*, aiming to intensify hyoscyamine production levels while preventing the formation of its derivatives, anisodamine and scopolamine. The sgRNA designed to target at the second exon within the ORF of the *Ab*H6H gene, regulated by the *At*U6-26 promoter, and the *Sp*Cas9 protein from *Streptococcus pyogenes* by the Cauliflower mosaic virus (CaMV35S) promoter. Seven plant lines out of the eleven transgenic plants examined in this study showed editing events characterized by frameshifts in the ORF region and changes to most amino acids, indicating a cumulative mutation rate of 63.6 percent (seven out of eleven) for the Cas-*H6H* system. Moreover, within the subset of these seven edited lines, three of the transgenic plant lines were observed to be homozygous, accounting for 27.3 percent (three out of eleven) of the total. The use of High-Performance Liquid Chromatography (HPLC) for analyzing alkaloids demonstrated the identification of a peak associated with hyoscyamine. However, no peaks related to anisodamine and scopolamine were observed in plants where the AbH6H-function had been disturbed (Zeng et al., 2021).

Three lines of *AbH6H* homozygous mutant roots exhibited a significant increase in hyoscyamine content with fold changes of 3.68-, 4.21-, and 4.28, respectively, compared to wild-type roots. With the specific goal of altering five target genes, CRISPR/Cas-based targeted mutagenesis was applied to the species *Dendrobium officinale*, targeting the enzymes *Coumarate 3-hydroxylase* (C3H), *Cinnamate 4-hydroxylase* (C4H), *4-coumarate coenzyme A ligase* (4CL), *Cinnamoyl coenzyme A reductase* (CCR) and *Irregular xylem5* (IRX) involved in the biosynthesis of lignocellulose. These enzymes are responsible for catalyzing various reactions within the pathway. It was observed that these enzymes can introduce alterations bordering on substitutions, insertions, or deletions in the generated products. The frequency of these edits can range from ten to a hundred percent. *Dendrobium officinale*, a highly regarded Chinese medicinal herb, possesses diverse pharmacological properties such as immunomodulation, anti-tumor effects, antioxidant activity, anti-fatigue properties, and renal protection, as demonstrated in experiments conducted on diabetic rats (Gao et al., 2002; Chen et al., 2014, 2021). Three sgRNAs were strategically designed at separate positions for every target gene. The sgRNAs in this study were governed by the OsU3 promoter, whereas the Cas9 enzyme, which incorporated plant-optimized codons, was controlled by the CaMV35S promoter. The mutagenesis targeting diverse gene candidates exhibited similar editing efficiency across all targets. One potential justification for this phenomenon could be attributed to variations in the chromatin structure at the specific genomic loci. Consequently, the accessibility of the Cas9 protein and the sgRNA is hindered in chromosome regions exhibiting a higher degree of compaction (Kui et al., 2017). To explore the potential applicability of CRISPR/Cas-based targeted mutagenesis in *Dioscorea zingiberensis*, a medicinal monocotyledon with a notable abundance of diosgenin in its rhizome was identified with the farnesyl pyrophosphate synthase gene (*Dzfps*) as a suitable target for the study. Diosgenin functions as a vital enzyme precursor in the synthesis of diosgenin, rendering it a significant subject of study in the context of numerous steroid hormone medications. Its pharmacological attributes encompass noteworthy potential in anti-inflammatory, anti-allergic, and anti-oxidant activities (Feng et al., 2018). The procedure involves the formation of E-isomer farnesyl pyrophosphate (*FPP*), preceding the facilitation of the stepwise condensation of dimethylallyl diphosphate (*DMAPP*) and geranyl diphosphate (*GPP*). The targeting sequence design for the sgRNA was aligned with the initial exon of the *Dzfps* gene. While the *Os*U3 promoter was responsible for regulating the expression of sgRNA, the CaMV35S promoter was the one that determined how much *Sp*Cas9 was produced. The observed target sites displayed five unique categories of changes, specifically deletions of 1, 4, 5, 8, and 10 base pairs. It is worth noting that all mutant versions exhibited chimeric mutations. All observed alterations led to frameshift mutations and premature termination codons. The observed reduction in squalene levels suggests that using CRISPR/Cas9 resulted in compromised functionality of *Dzfps*. The works, as mentioned earlier, provide sophisticated illustrations of merging CRISPR/Cas9 tool utility for conducting gene editing in medicinally-valued plants (Feng et al., 2018).

Knockout of genes by CRISPR/Cas9 often occurs through indel mutation, fragment deletion, domain, or large deletion generated by the NHEJ repair pathway, resulting in loss of function. This reverse genetics strategy is helpful for functional studies of a gene or transcription factor (TF) controlling any gene regulatory pathway. Knockout lines deleting important domains of a transcription factor leads to understanding whether it is a negative or positive regulator of a biosynthetic pathway. In *Salvia miltiorrhiza* knockout lines of the basic leucine zipper2 (bZIP2), gene was generated, precisely targeting the bZIP domain. The findings of this work indicate that bZIP2 exerts a suppressive influence on phenolic acid production. Shi et al. (2021) conducted a study on a novel bZIP member called bZIP2 in *Salvia miltiorrhiza* which also exhibited a robust response to the induction of abscisic acid (ABA) (Shi et al., 2021). Previous studies have provided limited evidence of the effectiveness of ABA in modulating the synthesis pathway of phenolic acids in *Salvia miltiorrhiza* hairy roots (Cui et al., 2012). Thus, the functional significance of bZIP family genes in the elevation of phenolic acid levels in *Salvia miltiorrhiza* is currently obscure due to minimal research on the regulating ABA-mediated modulation of phenolic acid synthesis.

Seventy bZIP TFs were examined using whole genome data from *S. miltiorrhiza*. These TFs were categorized into eleven sub-groups. Notably, the group-A bZIP TFs, specifically those belonging to the ABI-ABF-AREB subfamily, exhibited significant similarity to group-A members found in other plant species (Zhang et al., 2018a). The TFs as mentioned above are recognized as essential contributors to the ABA-dependent signaling cascade. Furthermore, Bhatnagar et al. (2017) made the observation that the group-A bZIP TFs exhibited four conserved regions (C1-C4) in association with a bZIP domain. The CRISPR/Cas9 vector harbors an *At*U6-sgRNA cassette engineered to target and disrupt the *bZIP2* gene. The CaMV35S promoter regulates this vector. A total of 45 transgenic hairy roots were produced, among which 18 samples exhibited insertions and deletions (indels) in the designated target region. The observed editing rate in these samples was determined to be 40 percent. The bZIP2 knockout lines displayed a significant elevation in phenolic acid content, with values ranging from 47.70 mg/g D.W. to 59.35 mg/g D.W. The observed increase exhibited a substantial escalation ranging from 23 to 53 percent compared to the unedited wild-type control (Shi et al., 2021). Among the various downstream genes examined, it was observed that the expression level of phenylalanine ammonia-lyase (PAL) exhibited the most notable increments in the knockout mutants. At the same time, while it decreased in the overexpression lines. The results connote that in *S. miltiorrhiza*, bZIP2 has a role as a suppressor in the phenolic acids biosynthetic pathway, specifically rosmarinic acid (RA) and salvianolic acid B (Sal B) (Shi et al., 2021).

**Figure 2:**
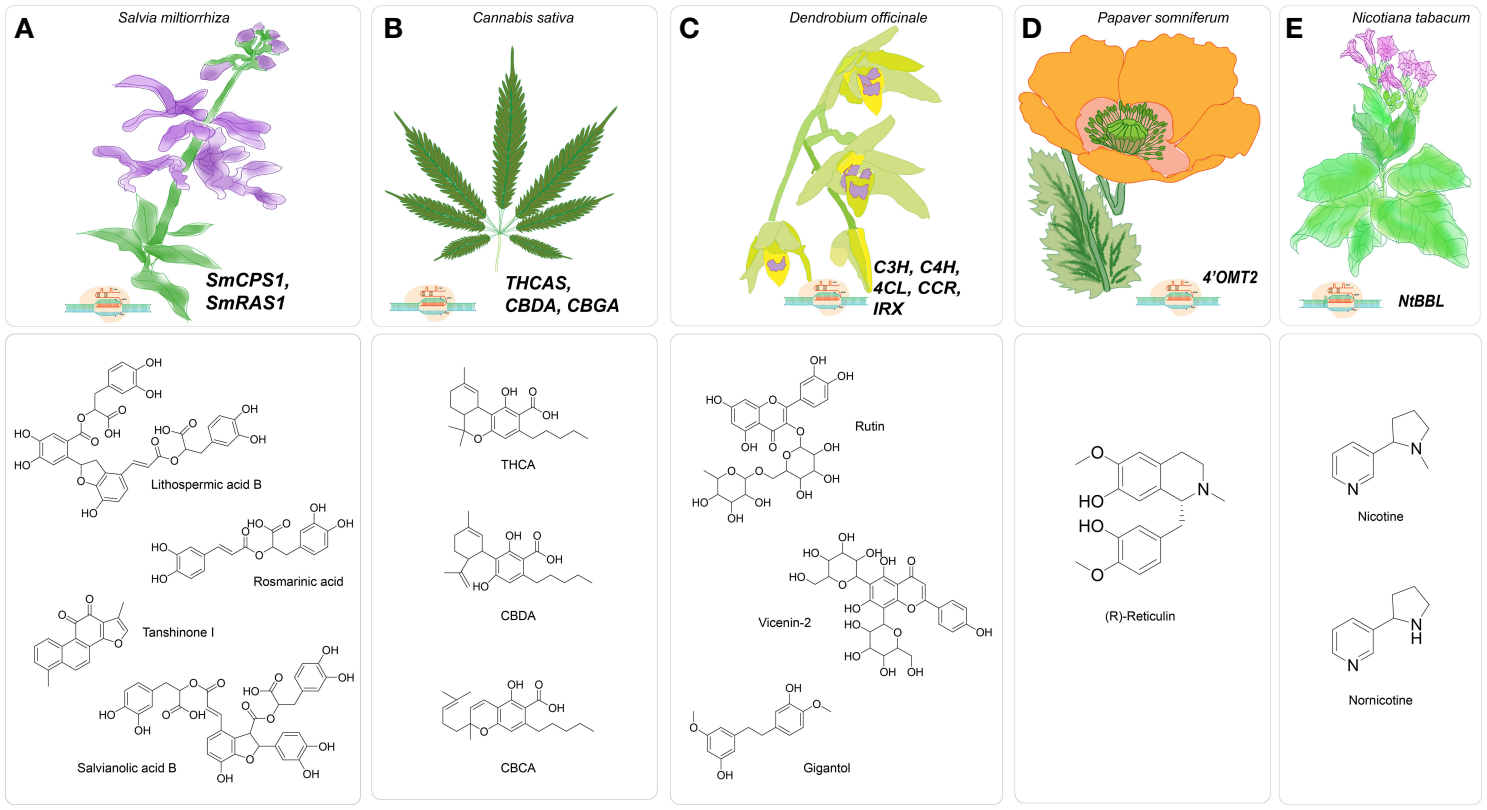
Major gene targets for CRISPR/Cas-mediated genome editing in medicinal plants **(A)**. CRISPR/Cas9-mediated genome editing in *Salvia miltiorrhiza* targeting *Sm*CPS1 and *Sm*RAS for enhancing specialized metabolites tanshinones, water-soluble phenolic acids, including rosmarinic acid, salvianolic acid B and lithospermic acid B; **(B)**. Proposed CRISPR/Cas9-based genome editing in *Cannabis sativa* targeting THCA, CBDA, CBGA to enhance cannabinoid production; **(C)**. CRISPR/Cas9- mediated gene editing in *Dendrobium officinale* targeting C3H, C4H, 4CL, CCR, IRX to enhance specialized metabolites bibenzyls of lignocellulosic pathway; **(D)**. CRISPR/Cas9-based genome *Papaver somniferum* targeting *Ps*4’OMT to improve synthesize of benzylisoquinoline, **(E).** CRISPR/Cas9-based genome editing in *Nicotiana tabacum* targeting *Nt*BBL for nicotine-free tobacco.

An additional illustration of a CRISPR-based knockout strategy is implemented to impair the biosynthesis of nicotine selectively assisted by flavoproteins affiliated with the berberine bridge enzyme-like (BBL) family. Previous research undertaken by Kajikawa et al. (2011; 2017) has revealed that six genes (BBLa, BBLc, BBLd.2) have their origins in *Nicotiana sylvestris*, while BBLb, BBLd.1, and BBLe have their origins in *Nicotiana tomentosiformis*. The research aimed was to identify homologous genes in *Nicotiana tabacum* and design sgRNA targeting a specific sequence that shares similarity with these genes, except for the PAM site (**Figure 2E**) (Kajikawa et al., 2011, 2017). The sgRNA was generated under the regulation of the *At*U6-26 promoter using the transformation vector pCas9-TPC. The vector utilized in this study contained a bar gene, which functioned as a marker for selection (Fauser et al., 2014). In the subsequent generations (T2 and T3), the nicotine level was determined to be 0.06 mg g per DW and 0.04 mg g per DW, respectively. The data mentioned above indicate a substantial decrease of 99.6 and 99.7 percent concerning to the wild-type. Notably, the T3 generation was determined to be largely lacking in nicotine (Schachtsiek and Stehle, 2019).

The CRISPR/Cas-based knockout technique was employed to investigate the regulation of benzylisoquinoline alkaloid (BIA) production in *Papaver somniferum*, a plant species known for its ability to biosynthesize morphine and BIAs utilized in the field of biomedicine (**Figure 2D**). The BIA biosynthesis commences with the amalgamation of dopamine and 4-hydroxyphenyl acetaldehyde (*4-HPAA*), resulting in the production of (S)-norcoclaurine (Fairbairn and Wassel, 1964; Valva et al., 1985; Ilari et al., 2009). The conversion of (S)-norcoclaurine into a key intermediate S-reticuline involves a sequence of methylation and hydroxylation reactions. The catalytic process is facilitated by 3’-hydroxy-N-methylcoclaurine 4’-O-methyltransferase (*4’OMT*) (Alagoz et al., 2016). Morphine, noscapine, and papaverine are final products that are obtained by distinct biosynthetic processes from S-reticuline. Previously, *4’OMT2* gene was overexpressed and subjected to TRV-mediated gene silencing experiments, but there is currently no conclusive evidence regarding its role in the synthesis of BIAs. Despite these studies, the biosynthetic pathway remains active, producing ongoing metabolite production. Consequently, the comprehensive phenotypic effects regulated by the targeted genes may be obscured (Gurkok et al., 2016). Therefore, knockout strategies utilizing a programmable CRISPR/Cas9 system effectively tackle the obstacles and elucidate the functions about the gene of interest. In this case, the 5’region of *4’OMT2* has been chosen as the target location for constructing a 20 base pair sgRNA. The viral expression vector system mediated by the Tobacco rattling virus (pTRV) was combined with the sgRNA, controlled by the AtU6 promoter, and the human-codon optimized version of Cas9 nuclease (hCas9), presided over by the CaMV35S promoter and nopaline synthase (nos) terminator. The resultant structure was injected into plant leaves via the agroinfiltration methodology. To assess the effectiveness of editing, the researchers utilized knockout lines to evaluate various compounds (morphine, S-reticuline, codeine, laudanosine, thebaine, noscapine, and papaverine) through the application of HPLC-ToF/MS and found their levels significantly reduced. The alkaloid accumulation, mostly thebaine and S-reticuline showed decreased levels for synthetic and viral-based CRISPR knockout lines (Alagoz et al., 2016).

In order to expedite the process of domesticating *Taraxacum kok-saghyz* (TK), commonly known as Rubber dandelion, a genetic element responsible for the production of *fructan-fructan 1-fructosyltransferase* (*1-FFT*) was targeted for disruption using the CRISPR/Cas9 approach. This gene contributes to the biosynthesis of inulin, a substance known to impair the production of rubber. *Taraxacum kok-saghyz* is recognized for its inherent capacity to synthesize rubber with a substantial molecular weight in its subterranean structures, rendering it a viable substitute for natural rubber. The selected site of the target for constructing sgRNA driven by the *At*U6-26 promoter was the second exon of the *1-FFT* gene due to its shared homology in all expected isoforms. Out of the eleven hairy roots, a significant proportion of ten were subjected to genome editing, demonstrating a mutation efficiency of 88.9%. According to Iaffaldano et al. (2016), it was observed that plants with genetically modified genomes were successfully regenerated from hairy roots in a time frame of six weeks (Iaffaldano et al., 2016).

One noteworthy report was conducted by Zhou et al. in 2018, wherein the researchers sought to clarify the functional importance of the rosmarinic acid synthase gene (*Sm*RAS) in the production of phenolic acids using the CRISPR/Cas9 approach (Zhou et al., 2018) (**Figure 2A**). A specific genomic locus, SMil_00025190, out of eleven homologous members in the RAS gene family, was selected from the *S. miltiorrhiza* genome database due to its notable expression across multiple organs. The sgRNA was strategized accordingly, to specifically binds with the targeted ORF of *Sm*RAS. The sgRNA utilized in this study was regulated by endogenous plant promoters, namely the *At*U6-26 derived from *Arabidopsis thaliana* and the *Os*U3 from *Oryza sativa*—conversely, the human-optimized coding sequence of h*Sp*Cas9 was modulated by the CaMV35S promoter. The mutation rate of the regenerated transgenic hairy roots was determined to be 50 percent based on the observations. A total of eight mutants, including two heterozygous mutants, two homozygous mutants, and five biallelic mutants, were present at the observed rate. The mutants derived from a cohort of 16 distinct transgenic hairy root lines, each induced by the sgRNA and regulated by the *At*U6 promoter. On the other hand, it was found that none of the 13 autonomous transgenic hairy root lines that were regulated by the sgRNA and under the control of the *Os*U3 promoter exhibited any mutant traits. Therefore, the proposal to identify appropriate synthetic or endogenous plant-specific promoters to regulate the expression of CRISPR/Cas9 has the potential to enhance gene editing efficacy. Additionally, the study of sixteen edited lines revealed a consequent decrease of ninety percent in the expression levels of the *Sm*RAS gene within the A8 homozygous mutant line, which is harbored a seven nucleotide deletion. Moreover, the other heterozygous and biallelic edited lines exhibited a reduction in expression levels ranging from around forty to eighty percent. Subsequently, metabolomics analysis showed a decrease in the phenolic acid content, specifically lithospermic acid B (LAB) and rosmarinic acid (RA), in homozygous mutants. When the potential target gene shows great similarity to other members of its gene family, the execution of functional analysis becomes more difficult. Consequently, investigating the of loss of function requires the knockout of multiple genes rather than just one. The *Sm*RAS gene, chosen as the potential target, is among the eleven members of the RAS gene family. The ten remaining RAS family genes continue to be involved in the transcription and translation, indicating that RA biosynthesis still takes place.

In their report published in 2022, Shiels et al. proposed implementing CRISPR/Cas system to target THC acid synthase gene (THCAS). This approach aims to create plants that are devoid of delta-9-tetrahydrocannabidiol (THC) but have high amounts of cannabidiol (CBD). The authors suggest that such plants could have increased commercial value, particularly in countries where there are strict regulations on THC content (Shiels et al., 2022) (**Figure 2B**). *Cannabis sativa* L., known as hemp, is a dioecious plant that has recently garnered significant attention due to its medical benefits, which have demonstrated economic viability. At present, the legalization of medical cannabis has been seen in over 50 nations, projecting a market worth of $20.2 billion within the timeframe of 2020-2025 (Aliekperova et al., 2020). The exploration of genome editing in *Cannabis sativa* remains an area that has not yet been thoroughly investigated, with the advent of improved the biosynthesis of cannabinoids and other specialized metabolites associated with various pathways. The increasing need for the creation and accumulation of cannabinoids presents an opportunity for the development of FDA-approved cannabidiol (CBD) and a potential reduction in the production of THC, which has been linked to various adverse consequences (Pertwee, 2008; Zuardi, 2008; Russo, 2011).

While omics analysis has identified multiple candidate genes within the cannabis production pathway that could potentially be targeted for genome editing, there is a scarcity of papers documenting successful and consistent transformation of cannabis tissues. Zhang et al. (2021) demonstrated an improvement in shoot regeneration efficiency by reprogramming of plant meristems, by overexpressing various genes, including Cannabis developmental regulator homologous to *Zm*WUS2, *Nb*STM, *Nb*IPT, *Os*GRF4, and *At*GIF, in immature embryo hypocotyls (Zhang et al., 2021). In order to develop albino-edited plants of Cannabis sativa, the CRISPR/Cas-mediated gene editing technique was employed, explicitly targeting the phytoene desaturase gene (*Cs*PDS1), commonly used as a marker gene. As a result, the *Cs*PDS1 gene knockout. According to Zhang et al. (2021), among the six sgRNA designs examined, sgRNA3, sgRNA4, and sgRNA5, which were specifically designed with Exon 6 of *Cs*PDS1, exhibited a greater editing frequency and efficiency compared to the other sgRNAs. These findings suggest the significance of the target site’s position in influencing the editing outcomes (Zhang et al., 2021).

In light of this, the CRISPR/Cas9 tool is highly efficient for gene editing, especially in reverse genetics studies intended to elucidate gene function by generating of knockout lines. However, when using this system, it is important to consider a number of important factors carefully, such as choosing the target locus, making a suitable sgRNA, and selecting the right promoters to drive the sgRNA. Moreover, the investigation of gene functionality presents increased complexity when the potential target gene for knockout pertains toa gene family member.

#### 3.2.2 Multiplex gene editing with CRISPR/Cas in medicinal plants

Multiplex editing of gene targets using CRISPR/Cas-based methods facilitates the investigation of genes that exhibit a high degree of redundancy. A more straightforward approach to studying the functional significance of a gene with numerous isoforms or gene family members in reverse genetics involves simultaneously targeting of several sgRNAs and Cas proteins inside a single expression vector system. When examining a gene family, it becomes crucial to investigate the functional role of an individual gene if it exhibits significant homology with other representatives of the gene family. Consequently, several genes should be knocked out to produce the desired phenotypic change; other gene family members could otherwise compensate. Zhou et al. (2021) conducted a study that employed CRISPR/Cas9 to operate a multiplex gene knockout to target the laccase gene family associated with *Salvia miltiorrhiza* by implementing a “dual-locus editing” strategy. *S. miltiorrhiza*, a plant commonly used for traditional medication in China, is perceived for its biologically significant lipid-soluble compounds, namely tanshinones, as well as water-soluble phenolics such as rosmarinic acid (RA), salvianolic acid B (SAB), and salvianic acid (Danshensu). These compounds demonstrated efficacy in cardio-cerebral and vascular disease treatments (Zhou et al., 2021). Laccases, classified as members of the benzenediol oxygen reductases (EC 1.10.3.2), are part of the multicopper-oxidase gene family. They are closely associated with the oxidation and polymerization of monolignols, suggesting their potential involvement in the synthesis of SAB (Festa et al., 2008). As of now, a total of twenty-nine laccases, referred to as *Sm*LACs, have been obtained from the *S. miltiorrhiza* database. Among them, *Sm*LAC7 and *Sm*LAC20 have been specifically investigated to assess their impact on the production of SAB (Li et al., 2019). Nevertheless, the evaluation of plant phenotypes cannot be adequately influenced by the functional role of a single *Sm*LAC gene due to the prolixity of redundant laccase genes (Simões et al., 2020). The sgRNAs utilized in the dual-locus editing approach were specifically intended to target the Cu-oxidase_3 domain and Cu-oxidase_2 domain. Hence, the entire ORFs of twenty-nine *Sm*LACs were aligned using the BLAST homology algorithm (Li et al., 2019). It was interpreted that the laccase genes in *S. miltiorrhiza* were organized into seven distinct clusters. The sgRNA1 specifically targeted a gene belonging to group VII, whereas the sgRNA2 targeted genes from groups I, II, III, IV, and V. The subsequent examination of phenolic acid metabolism revealed that *Sm*LACs have a significant outcome in altering lignin production, which is essential for both root development and phenolic acid metabolism. A significant reduction was observed in the expression of key genes associated with phenolic acids synthesis, indicating the potential involvement of *Sm*LAC genes in this biosynthetic process (Zhou et al., 2021).

*Nicotiana tabacum* has two enzyme residues, namely core α (1,3)-fucose (FucT) and core β (1,2)-xylose (XylT), recognized as plant-derived glycoproteins, responsible for inducing an immunological or allergic response. The strategy is to eliminate the presence of FucT and XylT to provide a secure and therapeutic method for producing recombinant proteins using a plant-based expression platform. Previous research studies by Strasser et al. (2008) in *N. benthamiana* demonstrate the application of RNA interference to silence the FucT and XylT to achieve this purpose (Strasser et al., 2008). A TALEN-based strategy was also employed to induce knockout mutations in the *Nb*FucT and *Nb*XylT in Nicotiana benthamiana (Li et al., 2016). However, using of RNAi and TALEN methodologies could not result in a comprehensive depletion of both enzymes due to the partial concurrent inactivation of multiple genes. Hence, a focused multiplex CRISPR/Cas9 system was constructed to deactivate many genes simultaneously. Thus, a multiplex knockout strategy was implemented using CRISPR/Cas9, explicitly targeting the conserved region of four *FucT* and two *XylT* genes in BY-2 cells of *Nicotiana tabacum*, generating a knockout line encompassing a total of 12 alleles, including isoforms. In order to enhance the probability of inducing mutations across all isoforms, the sgRNAs were directed to edit the highly conserved domains of the targeted genes specifically. The sgRNA designs were chosen to aim for the first exon of the *Nt*XylT gene and the third exon of the *Nt*FucT gene. These sgRNAs were assembled for multiplexing, with a tRNA placed after the U6-driven sgRNA that is located upstream of the polycistron and pcoCas9 enzyme presided over by the promoter CaMV35S (Xie, Minkenberg, and Yang, 2015). The western blot analysis of the total cellular proteins, employing antibodies that specifically recognize FucT and XylT, indicated no detectable signal in the two lines (11,12). This observation suggests that the gene inactivation process was successful and complete. The MALDI-TOP analysis for examining the N-glycans in samples 11 and 12 did not exhibit any detectable levels of FucT and XylT residues, in contrast to the wild-type control line. The control line showed that over eighty three percent carried FucT residues and ninety one percent of XylT (Mercx et al., 2017).

In 2017, Li et al. employed CRISPR/Cas9 to induce deletion in the diterpene synthase gene (*Sm*CPS1) responsible for tanshinone synthesis in *S. miltiorrhiza* (Li et al., 2018). Tanshinones are a class of lipid-soluble chemicals that is found to possess properties that enhance blood circulation and have anti-inflammatory effects. Tanshinones utilize geranylgeranyl diphosphate (GGPP) as the shared precursor in taxol production. The cyclization of GGPP into tricyclic olefin miltiradiene has been demonstrated by two diterpene synthases, namely *Sm*CPS1 and *Sm*KSL1. This finding underscores the possibility of redirecting the biosynthetic pathway of GGPP towards the production of diterpenes, such as taxol by inhibiting the flux of GGPP towards tanshinone. Previously, RNAi gene silencing of *SmCPS1* showed a decrease in tanshinone production. Nevertheless, it remained observable in chimeric mutants, suggesting that a significant occurrence of non-specific gene silencing occurs due to sequence homology, hence facilitating the degradation of transcripts that are not the intended targets (Cheng et al., 2014; Cui et al., 2015). A recent study by McCarty et al. (2020) demonstrates using a multiplexed CRISPR/Cas vector system to simultaneously express multiple sgRNAs or Cas enzymes (McCarty et al., 2020). Three specific exon locations were chosen within the coding region (first, fourth, and eleventh) of the *Sm*CPS1 gene. Consequently, the three sgRNAs were developed to be controlled by the *At*U6-26 promoter and the expression cassette for *Sp*Cas9 by promoter CaMV35S. The proportion of independent transgenic T0 lines exhibiting successful mutations in *Sm*CPS1 was approximately 42.3 percent. Consequently, out of twenty-six independent transgenic hairy root lines, three lines were identified as homozygous mutants, while eight lines displayed chimeric mutations. Subsequent metabolomics analysis utilizing advanced mass spectrometry techniques unveiled the absence of specific tanshinones in the homozygous mutant lines, notably cryptotanshinone, tanshinone I, and tanshinone IIA. Therefore, the utilization of CRISPR/Cas9 technology to create knockouts effectively inhibits the flow of metabolic processes involving GGPP, thereby highlighting the potential for redirecting GGPP towards the production of essential diterpenes such as taxol (Li, Cui, et al., 2018).

The plant known as Comfrey, scientifically classified as *Symphytum officinale*, possesses medicinal attributes characterized by anti-inflammatory and analgesic effects. In addition to possessing pharmaceutically useful metabolites, pyrrolizidine alkaloids (PAs) in tissues render it poisonous to humans. The CRISPR/Cas9-mediated knockout strategy successfully eliminated homospermidine synthase (*HSS*), which serves as the initial enzyme in the pathway of polyamine (PA) synthesis. The observed hairy root lines derived from the *hss* gene exhibited reduced homospermidine and PA levels upon the inactivation of only one *hss* allele. Furthermore, upon the inactivation of both alleles, no alkaloids were detectable. Three *At*U6-driven sgRNAs fabricated according to the target sites (exons three, seven, and eight) within the coding region of the *hss* gene were assembled along with *Sp*Cas9 driven by CaMV35S. The knockout lines showed no detectable PAs and 80% reduced levels of homospermidine, exhibiting that comfrey-derived economical phenolics such as rosmaniric acid and allantoin potentially be extracted from PA-free knockout lines without any lethal damage (Roeder et al., 2015; Zakaria et al., 2021). CRISPR/Cas-mediated engineering eliminates undesired multiple branching pathways by performing simultaneous gene knockouts by multiplexing (Alagoz et al., 2016; Sun et al., 2017). In the most recent study, six tomato genes were successfully deleted using 12 sgRNAs and a single CRISPR/Cas system expression cassette. Utilizing of an optimal technique to simultaneously target several target genes presents the potential for developing a highly efficient multiplex mutation system. For instance, the concurrent suppression of THCAS and CBDAs could be achieved to enhance the desired outcome (Čermák and Curtin, 2017; Deguchi et al., 2020).

### 3.3 Bottlenecks of using CRISPR/Cas for plant metabolic engineering

#### 3.3.1 Off-target effects

CRISPR/Cas system allows precise and effective modification of DNA sequences. However, off-target effects in similar gene sequences can limit its application (Kleinstiver et al., 2016; Modrzejewski et al., 2020). A logistic regression model identified five factors influencing off-target occurrence: (1) the number of mismatches and sgRNA structure; (2) mismatch position; (3) GC-content; (4) nuclease variants; and (5) delivery methods. Increasing mismatches reduces off-target effects, and optimizing sgRNAs minimizes them (Modrzejewski et al., 2020). The optimal design of a sgRNA aims to enhance the efficacy of targeting the desired genomic site while limiting any possible off-target effects. The off-target occurrence rates exhibit a reduction of up to fifty-nine percent when a single mismatch is present, and this reduction becomes even more pronounced when four or more mismatches are present. The mismatches present in the seed sequence, namely within the first eight nucleotides near the PAM site, have been found to reduce the event of off-target effects considerably. The current understanding of the impact of GC content, nuclease variations such as Cas9 nickases, and delivery techniques on off-target effects is finite (Doench et al., 2016; Kimberland et al., 2018; Shen et al., 2019; Chen, 2019b).

#### 3.3.2 The efficiency of CRISPR/Cas9 tool delivery

A significant obstacle to successful transformation is the effectiveness of CRISPR/Cas9 and sgRNA delivery to plant cells. The limited transformation efficiency and the challenge of selecting suitable plasmid vector systems hinder utilizing the CRISPR/Cas9 and sgRNA delivery system. There exist two strategies for surmounting the challenges. The initial approach involves Cas9 protein expression at the cellular level. Alternatively, a combination of Cas9-sgRNA can be prepared *in vitro* before the process of transformation or transfection, akin to the CRISPR ribonucleoprotein (RNP) system employed for protoplast delivery (as depicted in **Figures 1B, H**). An additional approach is the transportation of the sgRNA complexed with Cas9 protein to the nucleus through carrier nanoparticles (Dittmann et al., 2015; Tong et al., 2019).

#### 3.3.3 CRISPR Cas system driving tool machinery

Improvements are needed in the driving machinery of the CRISPR/Cas tool, including promoters, terminators, selectable markers, and transformation protocols, for its effective utilization in endogenous plant systems. The present constraints in the intrinsic regulatory mechanisms inside medicinal plants pose obstacles to the synthesis of valuable bioactive compounds and inhibit the progress of CRISPR/Cas-mediated editing efficacy (Zhou et al., 2018; Jiang et al., 2017; Tong et al., 2019).

#### 3.3.4 Linkage of phenotype to genotype

The gene editing CRISPR/Cas9 system provides both time and cost management advantages. However, linking the genotype to the phenotype poses a challenge in identifying the correct editing outcomes. To address this limitation, researchers have explored using CRISPR (MAGIC) systems and high-throughput biosensors for mapping genotypes and phenotypes (Lian et al., 2019).

#### 3.3.5 Effective approaches to transformation and regeneration

There is a requirement for establishing efficacious plant transformation and regeneration protocols of enhancing the efficiency of gene editing tools like CRISPR/Cas9 in various plant species. The limitation of not having an effective tissue culture or transformation system in several plant species has resulted in notable hindrances to attaining a high transformation efficiency. In order to address this constraint, the utilization of *in vitro* tissue culture and protoplast regeneration approaches assumes a pivotal role in enhancing the transformation and regeneration competence of the CRISPR/Cas9 approach (Zhang et al., 2013; Altpeter et al., 2016; Mout et al., 2017).

## 4 Implications for improvement of transformation technologies for efficient delivery of gene-editing reagents in medicinal plants

The CRISPR/Cas gene editing system has revolutionized the understanding of plant genomes, enabling comprehensive profiling of medicinally valuable specialized metabolites involved in various plant secondary metabolic pathways. Despite the CRISPR/Cas system’s efficiency in editing the plant genome, the bottlenecks and challenges slow down further improvement and application (Cardi et al., 2023). The challenges are greater when implementing gene editing tools in novel or recalcitrant medicinal plants to establish a genotype-independent standard protocol for *in vitro* tissue culture, transformation, regeneration, and mutation detection in regenerants. The improvement in conventional *Agrobacterium*-mediated transformation depends on T-DNA delivery to overcome low transformation efficiency and *in vitro* regeneration ability but results in stable integration and gene expression leading to genetically modified plants (Nadakuduti and Enciso-Rodriguez, 2021). As a result, it reduces the regulatory mandates for acceptance as gene-edited plants until the elimination of the transgene by the backcrossing procedure. It also allows for establishing alternative transgene-free approaches to derive gene-edited plants with reduced off-targets or establishing genotype-independent in planta transformation protocols that do not rely on *in vitro* tissue culture and *de novo* regeneration. However, these methods require further work in medicinal plant species to advance gene-editing delivery (Han et al., 2021; Che et al., 2022).

Here, we discuss advances in establishing *in vitro* cultures, including hairy roots, tissue, callus, and cell suspension, to deliver gene editing reagents. We propose further using the information for CRISPR/Cas-mediated engineering of secondary metabolite pathways in medicinal plants.

### 4.1 *In vitro* differentiated-adventitious hairy roots culture and plant tissue culture

*Agrobacterium rhizogenes* is frequently used to induce hairy root development in medicinal plants. This technique facilitates the investigation of gene functionalities and the augmentation of yields of specialized metabolites (Tian, 2015; Hidalgo et al., 2018; Mi et al., 2020). The hairy root disease is induced by the infiltration of soil bacteria into wounded areas of dicot plants, leading to foreign gene integration into the plant’s endogenous genome (Mi et al., 2020). Hairy root phenotypes are generated through T-DNA transfer in the root-inducing (Ri) plasmid, subsequent integration, and expression of a foreign gene from the Ri plasmid (Guillon et al., 2008). One of the most well-known effects of the gram-negative soil bacterium *Agrobacterium rhizogenes* is the development of hairy root disease. Hairy roots induced by *Agrobacterium rhizogenes* exhibit enhanced branching growth and differentiate from plagiotropic root development even without hormones, making them powerful tools in plant biotechnology (Srivastava and Srivastava, 2007). CRISPR/Cas9-mediated hairy root (HR) knockout lines of the homospermidine synthase (*HSS*) gene in *Symphytum officinale* were successfully generated by stable transformation using *Agrobacterium rhizogene*s (Zakaria et al., 2021). The resulting *hss* HR mutant line significantly reduced homospermidine and toxic pyrrolizidine alkaloids (PA) levels. CRISPR/Cas9 was also employed to generate hairy root mutant lines in *Atropa belladonna*, producing clinically significant tropane alkaloids, hyoscyamine, and scopolamine. For instance, an HR mutant line with a disrupted pyrrolidine ketide synthase (*PYKS)* gene, responsible for tropane skeleton construction, was developed to enhance generated pyrrolidine ketide synthesis (Hasebe et al., 2021).

*In vitro* plant tissue culture integrated with gene editing tool delivery, offers the distinct benefit of utilizing totipotent plant cells to derive chimeric regenerants and gene-edited plants, which can be employed to propagate rare, endangered plants (**Figure 1E**) (Rao and Ravishankar, 2002; Efferth, 2019; Cardi et al., 2023). Examples of CRISPR/Cas reagents delivery with *Agrobacterium*-mediated stable transformation via *in vitro* tissue culture methods are well established in a few medicinal plants, like *Atropa belladonna*, *Camelina sativa*, *Nicotiana tabacum* (Mercx et al., 2017; Jiang et al., 2017; Schachtsiek and Stehle, 2019; Zeng et al., 2021). Prospects to improve the transformation efficiency and *in vitro* regeneration to derive chimeric regenerants from edited cells for efficient gene editing in medicinal plants are still open to discussion (Cardi et al., 2023).

### 4.2 *In vitro* undifferentiated- callus and cell suspension culture

Plant-derived specialized metabolite production in microbial culture often suffers from erroneous post-translational modifications of proteins. Therefore, commercial plant suspension cell cultures have emerged as efficient and eco-friendly alternatives, successfully generating valuable metabolites. It is worth mentioning that there have been substantial advancements in genetic transformation and CRISPR/Cas-mediated genome editing in cell cultures of *Arabidopsis* and *Nicotiana tabacum*. Nevertheless, the idea of integrating gene editing tool delivery and cell cultures to manipulate heterologous biosynthetic pathways in medicinally-valued plants has yet to be widespread (Wu et al., 2021) (**Figure 1D**).

### 4.3 *Agrobacterium*-mediated transient transformation.

The cost-effective and time-efficient *Agrobacterium*-mediated transient transformation has gained interest for delivering gene editing reagents into plant systems because of the transient expression of Cas nucleases and deriving transgene-free gene-edited plant lines. Although polyethylene glycol (PEG)-mediated protoplast transformation for the transient delivery of CRISPR/Cas reagent is a rare example in medicinal plants. Few research studies show that there are initial attempts to establish *Agrobacterium*-mediated transient transformation in medicinal plants using reporter genes (Cardi et al., 2023). *Agrobacterium tumefaciens* injection and rapid *Agrobacterium*-mediated seedling transformation (FAST) were two novel techniques created by Xi and colleagues in 2020 to temporarily transform *Nicotiana benthamiana*, *Salvia miltiorrhiza*, and *Prunella vulgaris*. According to Xia et al. (2020), the expression of exogenous genes GUS and GFP was effectively achieved in *N. benthamiana* and *Prunella vulgaris*. However, the transient expression of exogenous genes in *S. miltiorrhiza* was hindered by a defense mechanism, resulting in the unsuccessful transformation of the seedling system using *A. tumefaciens* (Xia et al., 2020).

## 5 Major transcription factors family regulating the metabolic pathways in medicinal plants- potential gene editing targets for enhancement of specialized metabolites

Plants synthesize plant-specialized metabolites, inclusive of flavonoids, nitrogen-containing alkaloids, terpenoids, phenolics, and polyphenolic compounds, in response to both biotic and environmental stressors (Chezem and Clay, 2016; Erb and Kliebenstein, 2020). These metabolites are synthesized via many metabolic pathways encompassing enzymatic reactions and gene regulatory mechanisms. Transcription factors have an indispensable regulatory role in the biosynthetic pathways and augmentation of bioactive metabolites in plants (Patra et al., 2013). Transcription factors selectively bind to specific cis-regulatory elements in promoter regions, which affects gene expression and makes it easier for transcriptional complexes, which are active in the transcription process to form (Yang et al., 2012). The comprehension of gene regulation of the metabolic biosynthesis networks and the functional roles of transcription factors leads to the potential discovery or enhancement of novel specialized metabolites in plants (Xu et al., 2016). Gene editing TF genes for generating knockout lines helps to dissect their molecular function in a particular metabolic pathway. TFs with adequate genomic information in the database, with known functional regulatory roles in a specific biosynthetic pathway, can exponentially activate the production of specialized metabolites by applying CRISPR/Cas gene editing tools. Here, we emphasize the major TFs family regulating critical secondary metabolic pathways in medicinal plants, which can provide prospective ideas for choice of potential gene editing targets to enhance the level of specialized metabolites in medicinal plants.

For instance, Luis et al., 2013 studied the involvement of APETALA2/ethylene-responsive element binding factors (AP2/ERF) and WRKY TFs in the regulation of artemisinin production, a highly potent herbal remedy for malaria (Luis et al., 2013). The AP2/ERF family encompasses four distinct sub-families, namely the AP2, ERF, RA, and dehydration-responsive element-binding protein (DREB) subfamilies. The regulation and response of plant metabolism to biotic and abiotic stress conditions are governed by AP2 and ERF TFs. The upregulation of ERF TFs that can connect with amorpha-4, 11-diene synthase (ADS) and CYP71AV1 motifs (P450 monooxygenase) resulted in elevated levels of artemisinin in genetically modified *Artemisia annua* plants (Yu et al., 2012).

The WRKY family of transcription factors comprises several genes that possess WRKY domains consisting of sixty highly conserved amino acids. These transcription factors are part of a substantial group that performs various functions in higher plants. They are involved in regulating plant signaling pathways, controlling plant secondary metabolism, and improving plant resistance to biotic and environmental factors (Jiang et al., 2017). The overexpression of the WRKY gene resulted in a 1.8-fold increase in artemisinin content in transgenic *A. annua* plants compared to the control. This increase in artemisinin production was attributed to the upregulation of the CYP71AV1 motif by the WRKY transcription factors (Rushton et al., 2010).

Another transcription factor family, basic helix-loop-helix (bHLH), demonstrates significant involvement in metabolic pathways associated with bioactive substances such as terpenoids, iridoids, and seco-iridoids. These compounds possess anti-cancerous, anti-microbial, and anti-inflammatory properties (Xu et al., 2016). The bHLH TFs also play notable functional roles in several biological processes, including plant growth, development, regulation of phytohormone levels, and maintenance of homeostasis.

The leucine zipper gene family, known as bZIP TFs, are important in plant growth, physiological processes, and stress responses. These TFs are distinguished by a conserved bZIP domain, consisting of two distinct structural features. The first feature is an essential region responsible for DNA binding, while the second feature is a leucine (Leu) zipper dimerization region (Zhang et al., 2015). The elucidation of the functions performed by these transcription factors can enhance the synthesis of plant-specialized metabolites.

The MYB TFs, on the other hand, are distinguished by the presence of a conserved DNA-binding helix-turn-helix domain, as well as a highly varied C-terminal activation domain. The architecture of these entities are classified into four distinct types: 1R (consisting of R1/2, R3-MYB, and MYB-related domains), 2R (comprising R2R3-MYB domains), 3R (composed of R1R2R3-MYB domains), and 4R (comprising R1R2R2R1/2-MYB domains). Transcription factors have essential and diverse functions in development of plant tissue and organs and their growth. These responsibilities encompass the production of xylem, guard cells, trichomes, and root hairs, as well as the synthesis of specialized metabolites, namely, alkaloids, terpenoids, flavonoids, and phenolics. A study conducted by Li et al. (2022) highlighted the significance of MYB TFs in *Camelia sinensis*. For instance, *Cs*MYB8 and 99 regulated flavonoid biosynthesis (including catechins, anthocyanins, and flavonol), *Cs*MYB85 and 86 controlled caffeine production, *Cs*MYB9 and 49 influenced theanine synthesis, *Cs*MYB110 managed carotenoid production, *Cs*MYB68, 147, 148, and 193 played roles in mono-/sesquiterpenoid volatiles production, *Cs*MYB164 and 192 were involved in lignin synthesis, and *Cs*MYB139, 162, and 198 impacted the synthesis of indolic compounds (Li, Xia, et al., 2022).

## 6 Future directions and perspective towards implementation of CRISPR/Cas system for the enhancement of specialized metabolites in medicinal plants

Medicinal plants are important in various fields, such as pharmacology, biomedicine, nutrition, and industry. Engineering metabolic circuits by eliminating competitive or rate-limiting inhibitions to increase the production levels of plant-specialized metabolites are just a couple of the many possibilities for manipulating plant metabolic pathways. The CRISPR/Cas9 gene editing strategy shows promise in modifying target genes to meet commercial demands for enhanced metabolite production. Recent advancements in this technology have allowed for precise editing of the genome, epigenome, transcriptional regulation, and post-translational modifications. Advances in HDR for precision editing and chromosomal rearrangements driven by inversions or somatic cross-overs and base editing of single nucleotide have expanded the probability of adeptly using CRISPR/Cas technology.

Furthermore, CRISPR/Cas9 can generate large mutant libraries and enable gene knockout, knockdown (CRISPRi), and activation (CRISPRa). This technology can improve plant metabolic engineering by introducing DNA sequences from elite varieties, constructing new cell factories, and studying novel biosynthetic pathways. The ultimate objective is to increase the synthesis of pharmaceutically and commercially useful metabolites while decreasing the synthesis of harmful or undesirable compounds. Understanding the genetic, molecular, and biochemical processes is crucial for enhancing specialized metabolite accumulation in plant tissues. Various methods can be applied to enhance genome editing techniques, including using synthetic or endogenous promoters, terminators, and nuclear localization signals. Morineau et al. (2017) employed endogenous promoters from Camelina U3 and U6 to induce the production of Cas9 in a plant expression vector (Morineau et al., 2017). Therefore, the employment of CRISPR/Cas-mediated metabolic engineering approaches provide an opportunity to intensify the production of specialized metabolites in more significant quantities and unique configurations. Multiplex genome editing enables the concurrent manipulation of several genes, addressing the issue of excessive expression or suppression of individual genes or gene families. The emerging understanding of transcription factors and gene expression alterations holds promise for improvement in specialized metabolite production. The emerging understanding of transcription factors and gene expression alterations holds promise for improvement in specialized metabolite production.

The CRISPR/Cas system facilitates functional studies and enhances our understanding of novel pathways in medicinal plants, such as the cannabinoid and terpenoid pathways in *Cannabis sativa* (Bonini et al., 2018). Gracz-Bernaciak et al. (2021) propose applying the CRISPR/Cas methods to study latex production in laticiferous plants like *Chelidonium majus* L. and its antiviral activity (Gracz-Bernaciak et al., 2021). Establishment of efficient regeneration and stable transformation systems is of paramount importance in plant gene editing and the augmentation of specialized metabolites (**Figures 1B–E**). While the successful overexpression of endogenous genes using HDR remains a difficult task in medicinal plants, the utilization of CRISPR-based mutagenesis for endogenous gene modification has been widely established. Utilizing of cis-regulatory elements and untranslated regions (UTRs) presents various methodologies for precisely modulating gene expression. Recent studies have provided compelling evidence about the significant capabilities of manipulating cis-regulatory elements and untranslated regions (UTRs) in attaining meticulous regulation of gene expression. In brief, the CRISPR/Cas system is a gene editing technology that is both cost-effective and efficient, making it a promising candidate for further exploration in the field of medicinal plants (Shrestha et al., 2018; Wolter et al., 2019; Si et al., 2020).

## 7 Conclusion

The requirement for comprehensive genome and transcriptome data constrains the utilization of CRISPR/Cas9 gene editing. The acquisition of sequence data and the execution of functional investigations on metabolic pathways are paramount importance in the coordination of enzymatic activities and the regulation of branching metabolic pathways. Facilitating broader utilization of the CRISPR/Cas9 approaches in the context of therapeutic plants. This technological progress has the potential to facilitate the modification of essential genes responsible for some specific metabolite biosynthesis that have significant biological, nutritional, and pharmacological implications. The increasing understanding of genomes, transcriptomes, and proteomes associated with the production of the specialized metabolites, coupled with the progress made in gene editing technologies, presents an opportunity for the economically viable synthesis of these metabolites in medicinal plants of significant pharmaceutical importance. This potential development is approached within the context of regulatory and ethical frameworks.

## Author contributions

J-YK: Conceptualization, Funding acquisition, Supervision, Writing – review & editing. SD: Conceptualization, Methodology, Writing – original draft, Writing – review & editing. MK: Conceptualization, Funding acquisition, Supervision, Writing – review & editing.
